# Transcriptional cyclin-dependent kinases as therapeutic targets in bone metastasizing cancers

**DOI:** 10.3389/fonc.2026.1984003

**Published:** 2026-10-26

**Authors:** Tiina E. Kähkönen, Gergana Galabova, Michael Thormann, Jussi M. Halleen

**Affiliations:** OncoBone Therapeutics Ltd., London, United Kingdom

**Keywords:** bone metastases, cyclin-dependent kinase, drug target, metastatic disease, transcriptional CDKs

## Abstract

Cyclin-dependent kinases (CDKs) are established and emerging drug targets in cancer. Clinical success of CDK4/6 inhibitors in HR+/HER2- breast cancer has validated the CDK family therapeutically and stimulated development of both next-generation cell-cycle CDK inhibitors and inhibitors targeting transcriptional CDKs (CDK7-9, CDK11–13 and CDK19). Experience across these programs has shown that the therapeutic value of a CDK inhibitor is determined not only by kinase selectivity, but also by tumor dependency, compensatory pathways, normal-tissue biology, pharmacokinetic exposure and dosing schedule. Transcriptional CDKs are particularly attractive in cancers that depend on highly active oncogenic transcriptional programs, potentially creating a therapeutic window despite their essential roles in normal cells. In this review, we summarize CDKs as cancer drug targets with emphasis on transcriptional CDKs and metastatic disease. We specifically explore the potential of CDK9 inhibition in bone metastases, including effects on both tumor cell survival and RANKL-NF-κB -driven osteoclastogenic bone microenvironment, and discuss how CDK12 and CDK13 may provide complementary or context-dependent therapeutic opportunities.

## 1 Introduction to cyclin-dependent kinases

### 1.1 A short overview to CDKs

In humans, 21 CDKs have been identified (1, 2). CDKs are serine/threonine kinases that regulate diverse cellular processes, including cell cycle transitions and transcription. Cell cycle is divided into interphase, comprising G1, S and G2 phases, and mitotic (M) phase that are regulated by CDK2, 4 and 6 (G1), CDK2 (S), and CDK1 (G2 and M) (3–6). CDK activity is regulated by different cyclins that are important regulators of cell cycle (1, 3). D-type cyclins (cyclin D1, D2 and D3) bind to CDK4 and CDK6 and are essential for entry to G1 phase (7). Cyclin E binds to CDK2 and allows cell cycle progression from G1 to S phase (8). Once in S phase, CDK2 binds to cyclin A to initiate DNA replication (9). Cyclin A activity also contributes to late G2 phase together with CDK1 to promote entry to M phase (10). CDK1 regulates M phase by binding to cyclin B, completing cell division (11).

In addition to cell-cycle CDKs, several CDKs regulate transcription and are referred to as transcriptional CDKs, including CDK7-9, CDK11–13 and CDK19 (12). CDK8 and CDK19 with cyclin C have a regulatory role as part of the mediator kinase complex and can activate or repress transcription depending on promoter context, transcription factors and cellular state (13). CDK7, CDK9, CDK12 and CDK13 have direct and well-established roles in RNA polymerase II (RNA Pol II) transcription (**Figure 1**). CDK7/Cyclin H/MAT1 forms the CDK-activating kinase (CAK) module within TFIIH and phosphorylates Ser5 in the carboxy-terminal domain (CTD) of RNA Pol II, supporting transcription initiation and promoter clearance (1, 14, 15). CDK9 binds cyclin T to form positive transcription elongation factor b (P-TEFb), which phosphorylates Ser2 of RNA Pol II CTD as well as DSIF and NELF, thereby releasing promoter-proximal pausing and enabling productive transcriptional elongation (16, 17). CDK12 and CDK13 bind Cyclin K and regulate transcriptional elongation and RNA processing (18). CDK12 additionally supports transcription of genes involved in DNA-damage responses (19), whereas CDK11 has important functions in pre-mRNA splicing (20, 21).

**Figure 1:**
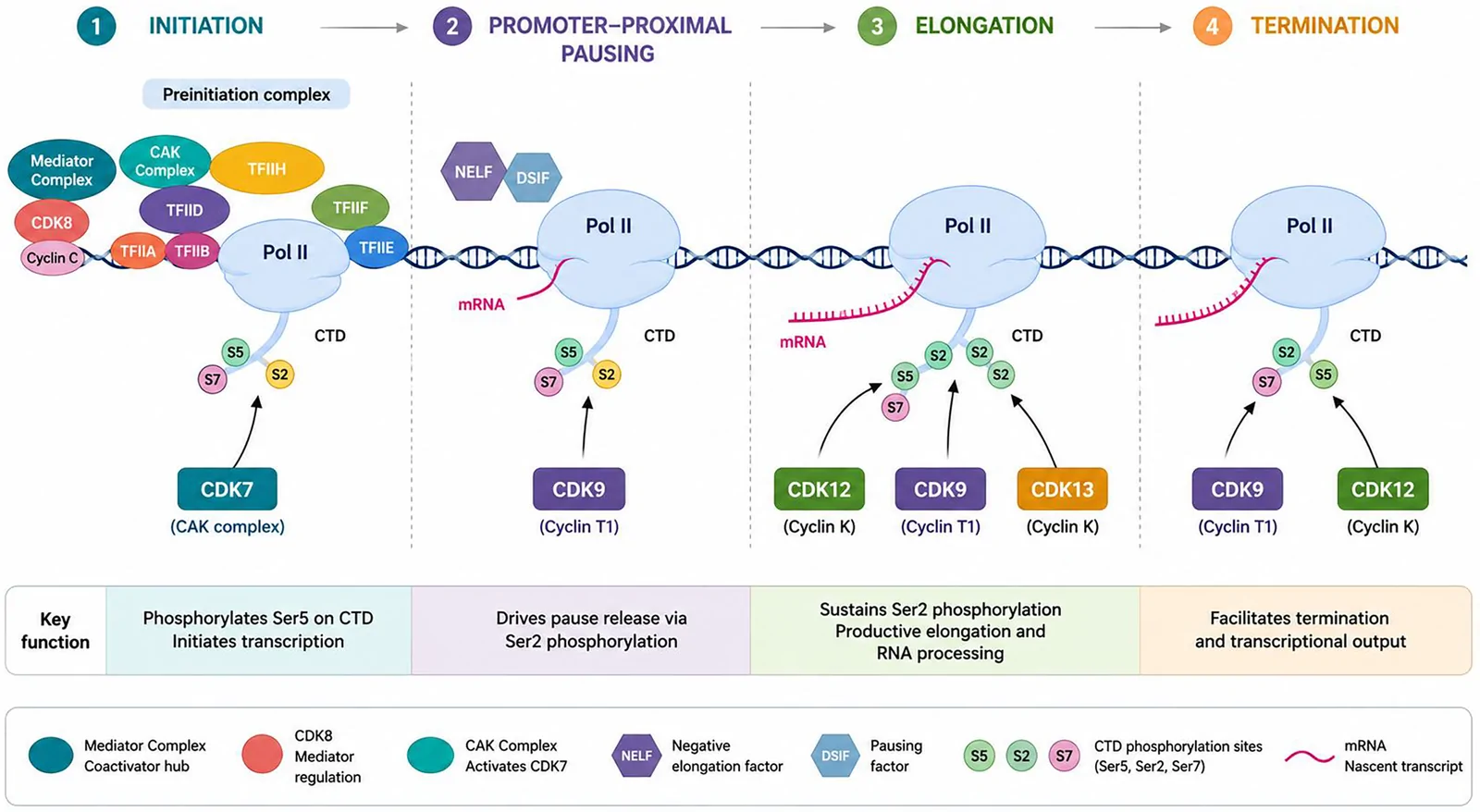
Function of transcriptional CDKs in regulating transcription from initiation to termination.

### 1.2 CDKs as drug targets in cancer

As dysregulated and uncontrolled proliferation is a hallmark of cancer and CDKs are one main class of proteins controlling cell cycle, CDKs have emerged as interesting drug targets in controlling tumor growth. Dysregulation and overexpression of several CDKs have been observed in cancer, suggesting that aberrant CDK activity may contribute to the uncontrolled proliferation (12). In addition to altered expression, mutations have been identified in genes encoding CDKs, cyclins, CDK-activating enzymes, CDK inhibitors, CDK substrates and checkpoint proteins, potentially contributing to tumor development and progression (3, 22–25). Furthermore, activity of CDKs and especially transition from G0 or G1 to S phase are regulated by extracellular signals. CDK activity, particularly during the G1/S transition, is regulated by mitogenic signaling pathways. Oncogenic pathways involving RAS–MAPK and PI3K–AKT–mTOR, as well as signaling through nuclear receptors such as the estrogen receptor (ER), can promote cell-cycle progression and are frequently dysregulated in many cancers (15).

Therapeutic potential of CDKs in cancer has been demonstrated clinically by the development and approval of the first CDK inhibitor palbociclib in 2015 by the FDA. Palbociclib inhibits CDK4/6 (26) and its success was subsequently followed by approval of ribociclib (27, 28) and abemaciclib (29) that are all used for treatment of hormone receptor (HR) -positive and human epidermal growth factor receptor 2 (HER2) -negative breast cancer as the first indication. The clinical success of CDK4/6 inhibitors has therefore validated CDKs as therapeutically relevant cancer targets and stimulated the development of inhibitors targeting other CDKs.

### 1.3 A short history and limitations of CDK drug development

More than three decades of CDK drug development have explored multiple CDKs as potential therapeutic targets across different cancer types (12). First-generation CDK inhibitors were relatively broad-spectrum and nonspecific, inhibiting multiple CDKs simultaneously and often referred to as ‘pan-CDK’ inhibitors (15). One such pan-CDK inhibitor flavopiridol inhibits cell cycle CDKs (CDK1, CDK2, CDK4 and CDK6) but also transcriptional CDKs (CDK7 and CDK9; 30). Flavopiridol induces cell-cycle arrest and inhibits transcription, resulting in potent anti-proliferative activity *in vitro*. However, its clinical activity in solid tumors was more limited (31–33). Finally, low activity of flavopiridol in Phase II trials in solid tumors led to program discontinuation (34–38). Clinical studies also revealed significant toxicities, highlighting the narrow therapeutic window associated with pan-CDK inhibitors.

Second-generation CDK inhibitors such as dinaciclib showed a narrower CDK inhibition profile than pan-CDK inhibitors like flavopiridol. Dinaciclib potently inhibits CDK1, CDK2, CDK5 and CDK9, with substantially lower activity against CDK4/6 and CDK7 (39). Dinaciclib showed broad anti-tumor activity *in vivo* in mouse models (40–43) and yielded promising Phase I results in patients with stable disease, and tolerable toxicity profile (44). However, Phase II results in solid tumors did not meet expectations and the program was discontinued (45, 46). Importantly, dinaciclib demonstrated greater clinical activity than flavopiridol in some settings, particularly in chronic lymphocytic leukemia (47). Although the difference may partly reflect distinct inhibition profiles of these two CDK inhibitors, this relationship has not been definitively established.

Later, CDK4/6 inhibitors were developed, approved and have become standard-of-care for HR+/HER2- breast cancer, particularly in advanced or metastatic setting where they are routinely given in combination with endocrine therapy. Since the approval of first CDK4/6 inhibitors in 2015, development of new CDK inhibitors has been rapid but is associated with some challenges that are discussed for example by Asghar and co-workers (15) and Galbraight and co-workers (13).

Challenges associated with CDK inhibitors include low specificity to certain CDKs, making it more difficult to clarify a detailed mode-of-action. Development of highly selective CDK inhibitors is challenging because CDK family members share considerable structural similarities, particularly within their ATP-binding sites (48). Studies of pan-CDK inhibitors have demonstrated that some CDKs may contribute more strongly to antitumor efficacy than others. For example, CDK9 has been shown to contribute to the antitumor activity of dinaciclib in triple-negative breast cancer (TNBC; 41). On the other hand, there is evidence suggesting that due to compensatory mechanisms between CDKs, specifically with CDK1, inhibition of more than one CDK (CDK1 and CDK2) is needed for efficient anti-proliferative effects and induction of apoptosis (CDK9) at least in NSCLC and osteosarcoma (49). There are also other studies suggesting that CDK1 and CDK2 contribute more to anti-tumor efficacy, while CDK4 has lesser effect in different human cancer cell lines (50).

Another major challenge in CDK drug development is identification and validation of biomarkers that can support patient selection and stratification in clinical trials, which can be an important cause of failure in later development. As indicated earlier, CDKs and their associated proteins can be overexpressed, downregulated or mutated in cancer, which can be used as selection criteria for clinical studies. Also, alterations in proteins indirectly associated with CDK activity, such as the oncogene MYC, have been reported to sensitize tumors to CDK inhibition (51). During the development of approved CDK4/6 inhibitors, it was noted that the greatest clinical benefit was observed in patients with HR+/HER2- breast cancer (52). Knowing the general response rates within the tumor indication or specific subgroups can further assist in patient selection. Another way to assist clinical development is to use biomarkers. MCL-1 has emerged as a potential pharmacodynamic and predictive biomarker for certain CDK inhibitors, particularly transcriptional CDK inhibitors. Indeed, MCL-1 levels are downregulated by CDK inhibitor treatment in preclinical models (53) and in clinical studies (54, 55). In addition to its potential relevance as a marker of response to CDK inhibition, MCL-1 expression has also been associated with tumor stage and disease progression (56).

### 1.4 Considerations for future CDK drug development

It is important to understand that clinical discontinuation of a CDK inhibitor does not necessarily indicate failure of the biological target itself, because limited clinical efficacy can have multiple pharmacological and biological causes. Limited efficacy may reflect inadequate target engagement, inability to achieve efficacious exposure due to dose-limiting toxicity, or a pharmacokinetic profile that does not provide sufficient duration of target inhibition. Clinical development of flavopiridol illustrates this concept, as optimization of the dosing schedule based on human pharmacokinetic data improved clinical activity, with responses correlating with unbound drug exposure (57, 58). Consequently, efficacy of transcriptional CDK inhibitors should be interpreted, whenever possible, in the context of free-drug exposure and pharmacodynamic target engagement rather than the administered dose alone.

Route of administration should also be considered together with pharmacokinetics and dosing schedule. Intravenous administration allows tightly controlled exposure profiles, whereas oral administration facilitates repeated, chronic or intermittent outpatient dosing and may be particularly practical for long-term treatment of metastatic disease. Oral administration is frequently preferred by patients, although adherence then becomes an additional determinant of exposure (59). For transcriptional CDK inhibitors, critical determinants of efficacy are therefore not the administration route itself, but free-drug exposure, peak concentration, duration of target inhibition, and recovery between doses. Clinical development of fadraciclib (formerly CYC065), transitioning from intravenous to oral administration, illustrates the importance of formulation and dosing schedule in achieving clinically feasible exposure with signs of activity at tolerable dose levels (60).

CDK drug development has shifted from broad kinase inhibition toward biologically informed selectivity. CDK inhibition profile of a compound can influence both efficacy and toxicity; increasing selectivity toward therapeutically relevant CDKs may reduce off-target toxicity and improve the therapeutic window. An important distinction, however, is between off-target toxicity caused by inhibition of unintended kinases and on-target toxicity resulting from inhibition of the intended CDK in normal tissues. Increased selectivity can reduce off-target toxicity, but it cannot eliminate on-target toxicity. For CDK9 in particular, the therapeutic window depends not only on kinase selectivity but also on tumor dependency, pharmacokinetic exposure, depth and duration of target inhibition, and dosing schedule (61).

A recent example of biologically guided kinase selectivity is atirmociclib (PF-07220060), which was developed with marked selectivity for CDK4 over CDK6. This strategy was based on biological dependency of HR+/HER2− breast cancer cells on CDK4, whereas CDK6 plays a critical role in hematopoiesis. By decreasing activity toward CDK6, atirmociclib reduced neutrophil toxicity while enabling greater CDK4 target coverage and improved antitumor activity in preclinical models (62). This illustrates that the therapeutic value of kinase selectivity lies in separating tumor-specific target biology from normal tissue.

In addition to improving efficacy and tolerability, kinase selectivity can be exploited to overcome adaptive resistance mechanisms. Activation of Cyclin E-CDK2 axis is a well-established mechanism for tumor cells to bypass CDK4/6 inhibition (63, 64). Consequently, selective CDK2 inhibitors such as INX-315, PF-07104091 and BLU-222 have emerged as a strategy to delay or overcome acquired resistance, providing preclinical evidence that CDK2 can become an actionable dependency in CDK4/6 inhibitor-resistant breast cancer models and that CDK2 inhibition can delay or reverse resistance (63–65). More recent translational studies further indicate that CDK dependency can shift from CDK4/6 toward CDK2 during resistance development (66). These findings illustrate that kinase selectivity can be used not only to improve tolerability but also to target adaptive bypass mechanisms created by prior therapy.

One potential therapeutic advantage of targeting transcriptional CDKs is that tumors with high transcriptional demand or dependence on oncogenic transcriptional programs may be more sensitive to partial suppression of transcription than normal tissues. Such differential dependency, rather than transcriptional inhibition per se, may provide a therapeutic window. Transcriptional CDKs may therefore be particularly attractive targets in cancers that exhibit high transcriptional activity and depend on oncogenic transcriptional programs, a phenomenon often described as oncogene addiction. MYC is one example of an oncogenic driver associated with increased transcriptional demand that has been investigated in the context of transcriptional CDK inhibition. Transcriptional CDK inhibitors in development have recently been reviewed by Kolodziejczyk and Sicinski (67).

### 1.5 Transcriptional CDKs as targets in cancer

To understand the relevance of transcriptional CDKs as therapeutic targets in cancer, it is important to determine in which cancer types their dysregulation is associated with tumor growth and progression. Breast cancer exhibits overexpression of CDK7, CDK8, CDK9, CDK11, CDK12 and CDK13 (67–69), while prostate cancer has dysregulated CDK7, CDK9 and CDK12 (70). Overall, elevated expression of transcriptional CDKs has been reported across at least 15 cancer types (67).

In general, high levels or activity of CDK7, CDK8, CDK12 and CDK19 are associated with tumor aggressiveness and poor clinical outcomes (67, 71). Unlike several other transcriptional CDKs, CDK9 is not consistently overexpressed across cancers. Instead, cancer cells may become dependent on CDK9-mediated transcriptional programs through oncogenic drivers such as MYC and expression of short-lived survival proteins such as MCL-1 (67, 72). Interestingly, alterations in CDK12 and CDK19 have also been associated with metastatic disease, although their functional contribution to metastasis remains less well understood. Interesting research in prostate cancer demonstrated that CDK12 loss during cancer progression induced synthetic lethality to CDK13 inhibition (73). CDK11 and CDK13 are among the least studied transcriptional CDKs. CDK10 appears to differ from several other transcriptional CDKs, as reduced CDK10 expression has been associated with increased invasiveness, metastasis and poor clinical outcome (74, 75).

Since the development of first and second generation CDK inhibitors, more selective inhibitors for transcriptional CDKs have emerged, including for example specific CDK7, CDK9 and CDK12/13 inhibitors (47). A search of ClinicalTrials.gov conducted on 24 August 2026 identified clinical studies evaluating selective CDK7, CDK8/19 and CDK9 inhibitors, whereas no clinical studies were identified for selective CDK11, CDK12 or CDK13 inhibitors. Among transcriptional CDKs, CDK9 has progressed furthest clinically, with multiple selective inhibitors entering or being evaluated in clinical trials (76).

Studies have indicated that dysregulation in CDK9 and CDK9-mediated pathways are related to tumorigenesis and tumor progression in multiple tumor types such as osteosarcoma (77), ovarian cancer (78), synovial sarcoma (79), pancreatic cancer (80) and endometrial cancer (81). Moreover, CDK9 expression or activation has been observed in several other sarcoma subtypes, including rhabdomyosarcoma, synovial sarcoma, Ewing sarcoma and chordoma, correlating with poor prognosis (82). In ovarian cancer, high CDK9 expression has been associated with tumor recurrence and distant metastasis. Functional studies further suggest a role for CDK9 in metastatic phenotypes as CDK9 inhibition reduces cancer cell migration and invasion (83). One potential mechanism underlying these effects is regulation of MMP9 expression by CDK9, because MMP9 contributes to extracellular-matrix degradation and thereby facilitates tumor cell invasion (84).

Although CDK9 is not a classical epigenetic enzyme, its activity is tightly linked to chromatin-dependent regulation of transcription. BRD4 recruits transcriptionally active P-TEFb to chromatin, thereby coupling histone acetylation to CDK9-mediated phosphorylation of RNA pol II and transcriptional elongation (85, 86). BRD4 can also recruit CDK9 through interactions with acetylated transcription factors such as RelA/p65, providing an additional link between chromatin-associated signaling and transcriptional elongation (87). This interaction is particularly important in cancers that have high dependence on oncogenic transcriptional programs. Consequently, CDK9 inhibition can disrupt transcriptional output of oncogenic chromatin states, including MYC-driven transcriptional programs, without CDK9 itself functioning as a canonical epigenetic modifier.

This becomes relevant especially because CDK9 is not commonly overexpressed in cancers, but CDK9 associated oncogenes c-myc and MCL-1 are. C-myc overexpression has been observed in many cancers but also related to disease progression and shown to induce Epithelial-to-Mesenchymal Transition in breast cancer (88), biochemical recurrence in prostate cancer (89), and progression and metastasis in colorectal cancer (90, 91). In fact, due to its oncogenic functions c-myc would be an interesting drug target, but it is considered ‘undruggable’ (92). Therefore, CDK9 inhibition may provide an indirect strategy for targeting MYC-dependent transcriptional programs, particularly in tumors exhibiting high MYC activity. Also, overexpression of the other CDK9 linked oncogene MCL-1 seems to correlate with poor prognosis and therapy resistance (93). In these cancers, CDK9 activity supports transcriptional programs involving oncogenic and pro-survival factors, including MYC- and MCL-1-associated pathways, which can contribute to tumor-cell survival and progression (82).

Preclinical studies suggest that CDK9 inhibition can suppress sarcoma-cell proliferation and tumor growth, in part through induction of apoptosis (79). The interferon-stimulated gene guanylate-binding protein 1 (GBP1) promotes PD-L1 expression through activation of CDK9 and STAT3 phosphorylation, thereby contributing to immune escape in osteosarcoma (94). These findings suggest that CDK9 may influence additional aspects of tumor microenvironment beyond direct regulation of tumor-cell proliferation and survival. CDK9 has also been detected in plasma-derived exosomes from breast cancer patients who developed resistance to CDK4/6 inhibitor treatment, suggesting a potential association between CDK9 and acquired treatment resistance (95).

Taken together, these findings link CDK9 to cancer progression and metastatic phenotypes, and raise the possibility that transcriptional CDKs may be therapeutically relevant in metastatic disease beyond their established roles in tumor cell proliferation and survival. Many CDK9 targeting inhibitors have been evaluated in preclinical and early clinical studies. In recent years, also other approached to affect CDK9 have emerged such as protein degraders, including PROTACs. PROTACs may offer more selective degradation of CDK9 over CDK9 inhibitors with prolonged cytotoxic effects (96). Some novel PROTACs have yielded promising results during preclinical characterization, including cytotoxic effects in lung, liver, bone and brain cancer and sarcoma cells (97). Furthermore, a CDK9 degrader has been shown to sensitize CRCP cells to PARP inhibition with synergistic activity, indicating a potential clinical combination (98). Whether transcriptional CDK inhibition provides a distinct anti-metastatic advantage over cell-cycle CDK inhibition remains to be determined. Although CDK4/6 inhibitors improve progression-free and overall survival in metastatic breast cancer, these clinical endpoints do not by themselves establish a metastasis-specific mechanism (99). Clinical observations also suggest that CDK4/6 inhibition may influence CNS metastatic progression, although such retrospective data do not establish a metastasis-specific mechanism (100).

In considering CDK biology in metastasis, CDK12 and CDK13 emerge as additional candidates of interest alongside CDK9. CDK12 and CDK13 share substantial sequence similarity, particularly within their kinase domains, but differ considerably in their less-conserved C-terminal regions (1, 12). Despite these similarities, both CDK12 and CDK13 regulate the expression of a distinct set of genes, suggesting shared but non-overlapping functions (1). Of the two, the functions of CDK12 are better understood than CDK13 (12).

CDK12 exhibits both context-dependent tumor-suppressive and tumor-promoting functions, as both loss-of-function and increased CDK12 activity have been described in different cancer contexts (1, 12). High CDK12 expression has been observed in 21% of primary unselected breast cancers, while 10% did not show any positive staining (101). CDK12 expression correlated with HER2 expression and the absence of CDK12 expression correlated with TNBC phenotype and reduced expression of DNA-repair proteins (1). CDK12 loss has also been suggested to uncover pharmacological vulnerabilities (73). Specifically, CDK12 regulates genes involved in DNA damage response and homologous repair, and pharmacological inhibition of CDK12 sensitizes cancer cells to PARP inhibition and other DNA-damaging therapies, providing a rationale for combination treatment strategies (12). CDK12 inactivation can promote genomic instability and increase the generation of neoantigens, potentially enhancing T-cell infiltration and creating an opportunity for immunotherapeutic strategies in immunologically cold tumors such as castration-resistant prostate cancer (CRPC, 102).

CDK13 amplifications and overexpression have been observed in primary hepatocellular carcinoma and colon cancer (103). Published data suggests that CDK12/13 inhibition has synergistic effects with DNA damaging drugs such as chemotherapy and PARP inhibitors (104), and CDK12/13 inhibitors may offer an advantage by targeting two processes, transcription and genomic instability, being important for survival and growth of many cancer cells.

Taken together, CDK12 and CDK13 should not be considered simply as linear downstream regulators following CDK9. Although all three kinases participate in transcriptional regulation, CDK12 and CDK13 have partially overlapping functions in transcriptional elongation, RNA processing, and regulation of DNA damage response genes, while controlling distinct transcriptional programs (18, 104). This creates a paralog redundancy between CDK12 and CDK13 where the other CDK may at least partly compensate for the function of the other, at least in genotype- and context-dependent manner. This paralog relationship has important therapeutic implications. In CDK12-wild-type tumors, functional overlap may permit compensation when only one kinase is inhibited, providing a rationale for dual CDK12/13 inhibition. In contrast, CDK12-deficient tumors exhibit increased dependency on the paralog CDK13, creating an opportunity for selective CDK13 inhibition through paralog-based synthetic lethality (73). Consequently, the optimal CDK12/CDK13 inhibition strategy is likely to depend on tumor genotype and biological context rather than kinase selectivity alone.

CDK12 and CDK13 remain less extensively explored as cancer drug targets. In a live-cell selectivity analysis, none of the profiled CDK inhibitors showed substantial activity against CDK12 or CDK13 (105), but more selective inhibitors and degraders have since emerged. SR-4835 is an ATP-competitive dual CDK12/13 inhibitor that demonstrates efficacy in TNBC patient-derived xenograft models (104). THZ531 is a covalent dual CDK12/13 inhibitor that has shown synthetic-lethal interactions with DNA-repair inhibition in multiple myeloma models (106). The covalent CDK12/13 inhibitor BSJ-01–175 demonstrates antitumor activity in Ewing sarcoma models (107), while another covalent CDK12/13 inhibitor YJZ5118 inhibits tumor growth in preclinical prostate cancer models (108). Degrader approaches have also emerged: BSJ-5–63 targets CDK12, CDK7 and CDK9 but not CDK13 and shows efficacy in prostate cancer models (109), whereas YJ9069 targets CDK12/13 and has demonstrated synthetic-lethal effects in prostate cancer models (110). Collectively, these compounds demonstrate growing interest in CDK12/13 as therapeutic targets while also illustrating that the desired selectivity profile depends on biological context.

CDKs have established roles in cancer cell proliferation, but their contribution to metastatic progression, particularly organ-specific metastasis, remains less well understood. **Figure 2** summarizes the functions, cancer relevance and therapeutic landscape of CDK9, CDK12 and CDK13.

**Figure 2:**
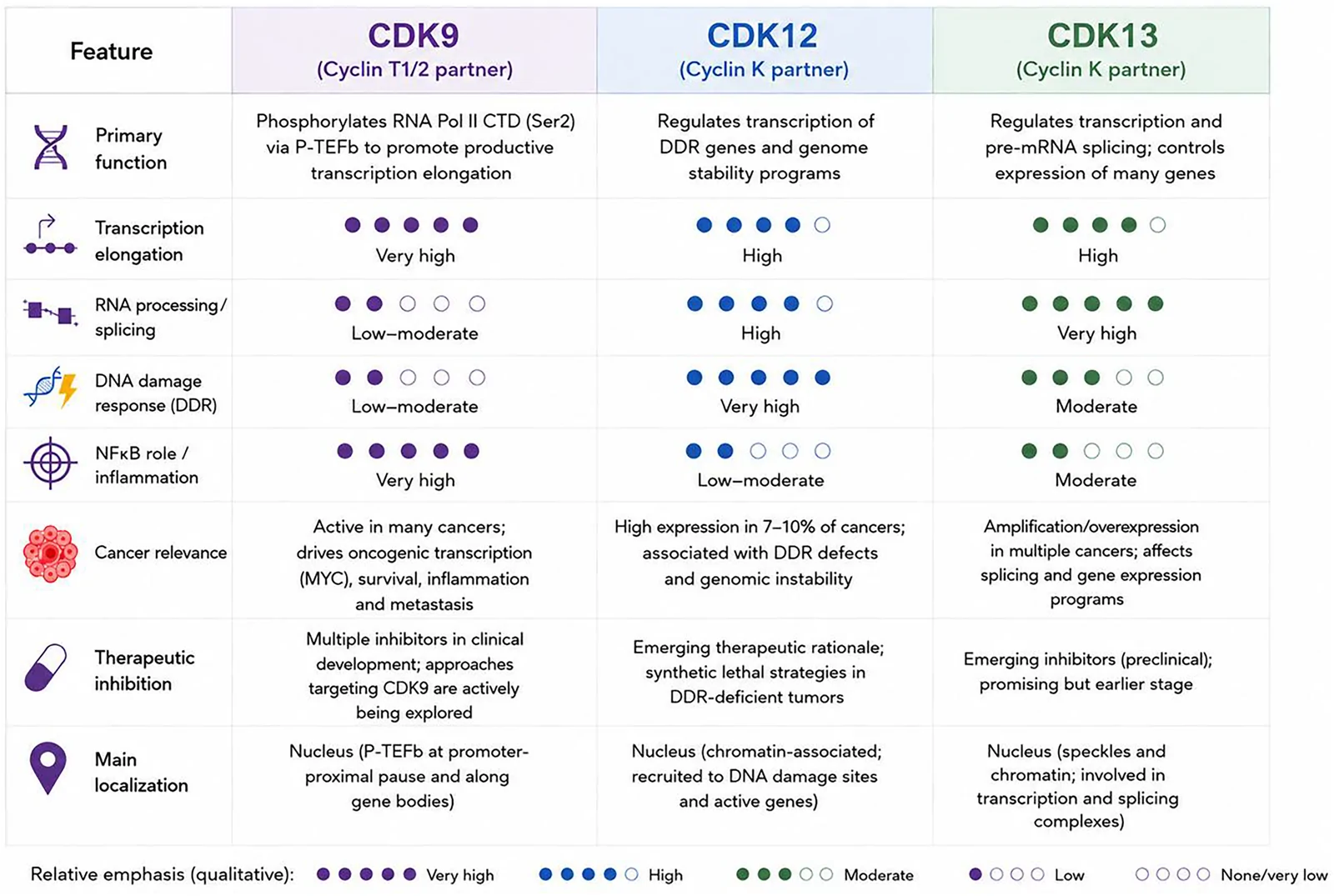
Summary of CDK9, CDK12 and CDK13 as individual and combined cancer targets.

## 2 Bone metastasis and CDKs

### 2.1 Clinical relevance and pathobiology of bone metastases

Bone metastases are a major clinical problem in several common cancers, particularly in breast and prostate cancer (111). The risk of bone metastasis is associated with advanced disease stage and aggressive tumor characteristics (112). In breast cancer, bone is the most common site of distant metastasis and approximately 70% of patients with metastatic disease develop bone metastases (113). Bone is also the first site of distant metastasis in approximately 25-40% of patients who develop metastatic breast cancer (112). Bone involvement is particularly frequent in advanced prostate cancer, where over 90% of patients with metastatic disease develop metastases to bone (114). Bone-homing or bone localized malignancies such as multiple myeloma and osteosarcoma, respectively, are biologically distinct from metastatic bone disease but the clinical complications are very similar. Approximately 80% of patients with multiple myeloma develop myeloma-related bone disease (115), whereas osteosarcoma is a primary bone malignancy with an estimated incidence of 2–4 cases per million people per year (116).

Bone metastases are rarely curable and therefore represent a major clinical challenge (117). One reason for the difficulty in treating bone metastases is the complex and highly tumor-supportive bone microenvironment. Importance of the bone microenvironment is consistent with Paget’s ‘seed-and-soil’ hypothesis, in which the properties of both metastatic cancer cells and the target tissue influence not only the formation but also the progression of metastases (117). Within bone, bone-resorbing osteoclasts, bone-forming osteoblasts, osteocytes, immune cells, and other stromal components interact with tumor cells to support metastatic growth. Tumor cells stimulate osteoclast-mediated bone resorption, releasing growth factors stored in the bone matrix that further promote tumor growth. This creates a self-reinforcing ‘vicious cycle’ in which tumor growth promotes bone destruction, which in turn facilitates further tumor growth (118).

Clinical consequences of bone metastases extend beyond uncontrolled tumor growth and include skeletal-related events (SREs) such as pathological fractures, spinal cord compression, hypercalcaemia, and the need of radiotherapy or surgery to bone (119). Cancer-induced disruption of bone homeostasis reflects an imbalance between osteoclast-mediated bone resorption and osteoblast-mediated bone formation. In breast cancer bone metastases, this balance is often shifted toward increased bone resorption, resulting in substantial bone loss (120), whereas prostate cancer bone metastases are frequently osteoblastic, with increased formation of pathological new bone (121). Treatment-related changes in bone metabolism may further contribute to skeletal complications in some patients (113). Taken together, these complications can substantially impair quality of life, particularly through severe cancer-associated bone pain (122).

New therapeutic strategies are needed for patients with bone metastases. An optimal therapy would ideally target both metastatic tumor cells and the pathological interactions between tumor cells and the bone microenvironment, thereby limiting tumor growth while preserving or restoring bone homeostasis and reducing the risk of SREs. Given the emerging evidence linking several CDKs to processes involved in cancer progression and metastasis, the potential role of CDKs as therapeutic targets in bone metastasis will next be considered, with particular focus on CDK9, CDK12, and CDK13.

### 2.2 CDKs as targets in bone metastases

As discussed above, CDK9 has been implicated in cancer progression and metastasis across multiple cancer types. In regards of connection to bone, high CDK9 expression has been observed in 67.1% of osteosarcoma patient samples where it was associated with shorter overall survival (77). In osteosarcoma, miR-206 has been identified to interact with MALAT1, which can further regulate osteosarcoma progression by modulating CDK9 expression (123). Another miRNA affecting osteosarcoma progression and formation of metastases via CDK9 is miR-874 that directly targets CDK9 and suppresses its expression, resulting in reduced proliferation and invasion of osteosarcoma cells (124). CDK9-associated non-coding RNA regulatory networks have also been implicated in other cancers that can metastasize to bone, including bladder cancer. In fact, GAS6-AS2/miR-298/CDK9 axis has been demonstrated to increase proliferation and metastasis, whereas knockdown of these proteins reduces tumor growth and metastasis *in vivo* (125). miRNAs may therefore have an important role regulating CDK9-mediated progression and formation of metastases.

Patients with bone metastases suffer from two related but distinctive phenomenon. First, uncontrolled tumor growth that could be regulated by CDK9 inhibition of tumor growth -promoting transcriptional activity. Secondly, tumor-cells promote activity of bone resorbing osteoclasts, eventually leading to development of SREs. These are linked but separate consequences of bone metastasis that are currently treated with different therapies but could be treated with one therapy with a dual function of anti-tumor and bone loss inhibiting effects.

Interestingly, CDK9 has been linked to bone and bone-related diseases outside of cancer. CDK9 is highly active in *in vitro* and *in vivo* models of inflammation, such as in osteoarthritis models. CDK9 inhibitor LDC067 reduces inflammatory responses and protects articular cartilage in osteoarthritis models by suppressing inflammatory cytokines and matrix metalloproteinases, including MMP3, MMP9 and MMP13 (126). Similar findings have been found in a post-traumatic osteoarthritis model where CDK9 inhibiting flavopiridol-loaded microparticles resulted in significant joint retention, decreased MMP activity as an indicator of joint inflammation 3 days post-injury, and reduced osteoarthritis disease severity at 28 days (127). Importantly, CDK9 inhibition has also been directly linked to regulation of bone resorption. Flavopiridol suppresses bone resorption in both titanium particle-induced osteolysis and ovariectomy-induced osteoporosis models and inhibits Receptor Activator of Nuclear factor-κB Ligand (RANKL)-induced osteoclastogenesis *in vitro* (128).

CDK9 inhibition reduces RANKL-induced phosphorylation and nuclear activation of p65/NF-κB and suppresses expression of downstream osteoclast differentiation and effector markers, including tartrate-resistant acid phosphatase (TRACP) and cathepsin K (CTSK), providing a mechanistic basis for reduced osteoclastogenesis and bone resorption (126, 128). RANKL should therefore be considered an upstream osteoclast differentiation signal, NF-κB and NFATc1 key signaling/transcriptional regulators, and TRACP and CTSK downstream markers of osteoclast differentiation and function. CDK9 has also been linked to transcriptional regulation of BMP and TGF-beta pathways, which are important in bone remodeling (129).

RANKL-driven osteoclastogenesis provides a direct mechanistic link between CDK9-dependent transcription and bone metastatic microenvironment. Following RANK activation, NF-κB signaling induces osteoclastogenic transcriptional program through activation of c-Fos and NFATc1, leading to expression of osteoclast differentiation and effector genes including TRACP, CTSK and DC-STAMP (126). P-TEFb/CDK9 contributes to productive transcription of NF-κB-responsive genes, while acetylated RelA/p65 recruits BRD4, which in turn recruits CDK9 to promote RNA pol II -dependent transcriptional elongation (87). Consistent with this mechanism, pharmacological CDK9 inhibition suppresses RANKL-induced NF-κB signaling, osteoclast differentiation and bone resorption, whereas the selective CDK9 inhibitor LDC000067 inhibits NF-κB/NFATc1 signaling and protects against osteolysis *in vivo* (126, 128).

More recent preclinical studies further support the therapeutic potential of selective oral CDK9 inhibition in metastatic disease. ORI-15412, a selective oral brain-penetrant CDK9 inhibitor, reduces metastatic burden across brain, bone, bone marrow, lymph nodes and lung in an intracardiac MV4–11 acute myeloid leukemia model, while suppressing phosphorylation of RNA Pol II, MCL-1 and c-MYC, inhibiting osteoclast differentiation, and reducing NF-κB-dependent p52 expression. Importantly, IND-enabling safety and toxicity studies identified no adverse events at efficacious doses, providing preclinical evidence that selective CDK9 inhibition can achieve an antimetastatic therapeutic window (130). Additional studies with ORI-15412 demonstrated oral antitumor activity, reduced PD-L1 expression and enhanced efficacy in combination regimens in melanoma and TNBC models (131). OBP-004, described as a dual inhibitor of CDK9/13, reduces metastatic tumor burden in bone, brain, lung and lymph nodes in MV4–11 model and inhibits TNBC growth in bone (132). Furthermore, OBP-004 extends these findings to CRPC bone metastasis model, with reductions in metastatic growth, cancer-induced bone loss and bone pain (133). Both inhibitors have demonstrated anti-tumor activity across different metastases, with profound efficacy on bone metastases. Collectively, these data support a model where transcriptional CDK inhibition can simultaneously target tumor-cell survival and pathological remodeling of bone microenvironment.

Taken together, these findings suggest two potential mechanisms through which CDK9 inhibition could influence bone metastasis. First, CDK9 inhibition may directly suppress tumor cell proliferation, invasion and survival, thereby reducing the ability of cancer cells to establish and maintain growth of metastatic lesions in bone, but also in other metastatic sites. Second, CDK9 inhibition may influence bone microenvironment by suppressing osteoclastogenesis and pathological bone resorption.

Preclinical reduction of metastatic tumor burden should be distinguished from prevention of metastatic dissemination, as these assess different aspects of metastatic biology. Clinical translation is further complicated because bone lesions without a measurable soft-tissue component are non-measurable under RECIST 1.1, and iRECIST retains the RECIST 1.1 framework for lesion measurability (134, 135). Future studies should therefore incorporate bone-specific endpoints, including skeletal-related or symptomatic skeletal events, imaging, biomarkers and pain assessment, to evaluate therapeutic effects within the bone microenvironment. However, in prostate cancer, the Prostate Cancer Clinical Trials Working Group 3 (PCWG3) provides disease-specific recommendations for assessing cancer-related bone disease (136).

To date, to our knowledge, no clinical study has demonstrated that CDK9 inhibition specifically improves outcomes of bone metastases. This may partly reflect limited bone-specific evaluation in trials of CDK9 inhibitors, even in indications in which bone metastases are frequent, such as TNBC and CRPC. It also remains possible that CDK9 inhibition alone may be insufficient to control all components of bone metastatic disease and associated skeletal complications.

If CDK9 inhibition alone is insufficient to control all components of bone metastatic disease, other transcriptional CDKs become relevant to consider. In prostate cancer, CDK12 alterations have been associated with aggressive clinical features and a shorter time to development of metastases. In one cohort, patients with CDK12-mutant prostate cancer had a median time to metastasis of 34.9 months, significantly shorter than patients in other genomic subgroups (71). Evidence linking CDK13 specifically to bone metastases remains limited. However, preclinical studies of dual CDK9/CDK13 inhibition in TNBC and CRPC bone metastasis models have reported reduced tumor growth, bone loss and bone pain (133).

These observations suggest that targeting CDKs beyond CDK9 may add therapeutic benefit through complementary biology rather than through a simple linear blockade of successive steps in the transcription cycle. CDK12 and CDK13 are paralogous Cyclin K -associated kinases with partly overlapping functions. Their dual inhibition may therefore prevent functional compensation in CDK12-wild-type tumors, whereas CDK12 loss can create a selective dependency on CDK13 (73). CDK12/13 inhibition can also impair DNA-damage-response programs and sensitize tumor cells to DNA-damaging therapies, including PARP inhibitors (101). This is clinically relevant because PARP inhibition has demonstrated efficacy in BRCA1/2- or other homologous-recombination-repair-deficient metastatic prostate cancer, including clinically relevant effects on progression and skeletal outcomes (137). Together, these findings provide a rationale for testing transcriptional CDK combinations in bone metastatic disease, while the optimal target profile should be defined by tumor genotype, pathway redundancy, exposure and tolerability.

## 3 Summary and conclusions

Bone metastases remain a major unmet medical need in several common cancers, particularly in breast and prostate cancer. This review highlights a broader lesson from CDK drug development: the therapeutic value depends on a biologically appropriate target profile, exposure and dosing schedule rather than on maximal kinase selectivity alone. Selective CDK4 inhibition can spare CDK6-dependent hematopoiesis, selective CDK2 inhibition can address adaptive resistance to CDK4/6 inhibitors, and CDK12/CDK13 targeting illustrates how paralog redundancy or synthetic lethality can favor either dual or genotype-selective inhibition. Within bone metastatic disease, CDK9 is particularly compelling because it connects oncogenic transcriptional dependency with the bone microenvironment. CDK9/P-TEFb inhibition can suppress RNA Pol II -dependent survival programs such as MYC and MCL-1, and through BRD4/NF-κB-linked transcription interfere with RANKL-driven osteoclastogenesis and pathological bone resorption. CDK9 inhibitors have not only demonstrated strong anti-tumor efficacy in preclinical studies without observed adverse events, but also reduced bone loss and pain, which is further supported by findings in cancer-independent preclinical models of bone loss. Future clinical validation will require bone-specific endpoints, because conventional response criteria incompletely capture skeletal disease. Taken together, transcriptional CDKs represent a mechanistically differentiated strategy to target both metastatic tumor cell dependencies and tumor-supportive bone niche, with future development guided by tumor genotype, pharmacodynamic target engagement, exposure and tolerability.
